# Bowel-Related Symptoms and Dietary Fiber Intake in Colorectal Cancer Survivors

**DOI:** 10.1001/jamanetworkopen.2025.42147

**Published:** 2025-11-10

**Authors:** Niels Klaassen-Dekker, Ben J. M. Witteman, N. Tjarda van Heek, Flip M. Kruyt, Eva Valgaeren, Elske C. Gootjes, Heidi Rütten, Pieter van ‘t Veer, Renate M. Winkels, Fränzel J. B. van Duijnhoven, Johannes H. W. de Wilt, Ellen Kampman, Dieuwertje E. Kok

**Affiliations:** 1 Division of Human Nutrition and Health, Wageningen University & Research, Wageningen, the Netherlands; 2 Department of Gastroenterology and Hepatology, Gelderse Vallei Hospital, Ede, the Netherlands; 3 Department of Surgery, Gelderse Vallei Hospital, Ede, the Netherlands; 4 Department of Dietetics, Gelderse Vallei Hospital, Ede, the Netherlands; 5 Nutrition and Healthcare Alliance, Gelderse Vallei Hospital, Ede, the Netherlands; 6 Department of Medical Oncology, Radboud University Medical Center, Nijmegen, the Netherlands; 7 Department of Radiation Oncology, Radboud University Medical Center, Nijmegen, the Netherlands; 8 Department of Surgery, Radboud University Medical Center, Nijmegen, the Netherlands

## Abstract

**Importance:**

Colorectal cancer (CRC) survivors frequently report bowel-related symptoms, but longitudinal data are scarce and diet has not been extensively investigated in relation to bowel-related symptoms.

**Objective:**

To investigate the prevalence of bowel-related symptoms as well as their association with dietary fiber intake until 5 years after CRC diagnosis.

**Design, Setting, and Participants:**

This prospective cohort study using data from the Colorectal Cancer: Longitudinal, Observational Study on Nutritional and Lifestyle Factors (COLON) study included CRC survivors with stage I-IV disease recruited at diagnosis from 11 hospitals in the Netherlands between August 2010 and February 2020. CRC survivors were followed up until 5 years after diagnosis. Data were analyzed between April 2024 and March 2025.

**Exposures:**

Clinical data including CRC treatment strategy, retrieved from hospital records and national registries, and habitual dietary fiber intake, assessed by a food frequency questionnaire, at 6 months, 2 years, and 5 years after CRC diagnosis.

**Main Outcomes and Measures:**

Prevalence of self-reported diarrhea, constipation, flatulence or bloating, frequent stools, mucus in stools, or false urgency at 6 months, 2 years, and 5 years after diagnosis, obtained via a questionnaire. The validated European Organisation for Research and Treatment of Cancer Quality of Life Questionnaire Core 30 (EORTC QLQ-C30) was also used to evaluate prevalence of moderate-to-severe diarrhea or constipation and health-related quality of life (HRQOL) at these time points. Odds ratios (ORs) for associations between fiber intake and bowel-related symptoms (yes or no) were calculated using multivariable logistic regression.

**Results:**

Among 1751 CRC survivors who underwent surgery and were included in the analysis, median age at diagnosis was 66 years (IQR, 61-71 years), and 1115 (63.7%) were men. Bowel-related symptoms were reported by 817 of 1751 survivors (46.7%) at 6 months, 614 of 1511 (40.6%) at 2 years, and 290 of 812 (35.7%) at 5 years after diagnosis. At 6 months after diagnosis, symptoms were predominantly reported by survivors who had received chemotherapy (260 of 446 [58.3%]), whereas symptoms at 2 and 5 years occurred mostly in those who received radiotherapy (86 of 160 [53.8%] at 2 years and 47 of 92 [51.1%] at 5 years). Of the studied bowel-related symptoms, diarrhea was associated with the lowest HRQOL score (B, −9.6; 95% CI, −14.0 to −5.2) at 5 years after diagnosis. In multivariable logistic regression analyses, higher fiber intake (per 10-g/d increment) was associated with a significantly lower prevalence of moderate-to-severe diarrhea at 6 months (OR, 0.44; 95% CI, 0.28-0.70) and 2 years (OR, 0.53; 95% CI, 0.30-0.94) after diagnosis, but the association was not statistically significant at 5 years (OR, 0.43; 95% CI, 0.16-1.13).

**Conclusions and Relevance:**

In this cohort study of CRC survivors, the prevalence of bowel-related symptoms was considerably high during the 5 years after diagnosis, highlighting a need for effective symptom management. Higher dietary fiber intake was associated with a lower prevalence of diarrhea, suggesting a potential role of diet in management of bowel-related symptoms in CRC survivors.

## Key Points

**Question:**

What is the prevalence of bowel-related symptoms and the association between dietary fiber intake and these symptoms up to 5 years after colorectal cancer (CRC) diagnosis?

**Findings:**

In this cohort study of 1751 CRC survivors, the prevalence of bowel-related symptoms was 47% at 6 months, 41% at 2 years, and 36% at 5 years after CRC diagnosis. Higher dietary fiber intake was associated with a lower prevalence of diarrhea.

**Meaning:**

The finding of prevalent long-term bowel-related symptoms in CRC survivors suggests a need for symptom management, with a potential role for dietary fiber intake.

## Teaser

This cohort study examines the prevalence of bowel-related symptoms up to 5 years after a colorectal cancer diagnosis and whether dietary fiber intake is associated with symptom prevalence.

## Introduction

Colorectal cancer (CRC) survivors frequently report bowel-related symptoms, such as diarrhea, higher urgency or frequency of stools, constipation, or bloating.^1,2,3,4^ Systematic literature reviews have summarized evidence regarding bowel-related symptoms^5,6^ with a focus on symptoms related to low anterior resection syndrome.^5,7,8^ Longitudinal data on bowel-related symptoms in CRC survivors and comparisons across various treatment modalities are scarce. Such studies are essential to gain insight into patterns of bowel-related symptoms over time and identify which survivors are at highest risk of those symptoms.^6,9^

Some factors associated with bowel-related symptoms, such as rectal tumors, receiving radiotherapy, or low anterior resection, are well established.^5,6,10,11^ However, little is known about the potential role of lifestyle in relation to bowel-related symptoms. Lifestyle adjustments, including dietary changes, tend to be welcomed by CRC survivors to manage their bowel-related symptoms, but to our knowledge, no evidence-based interventions are available.^12,13,14^ CRC survivors often report that they increase intake of dietary fiber as a self-care strategy to manage bowel-related symptoms.^15,16,17^ Furthermore, higher fiber intake has been associated with improved stool patterns and fewer bowel-related symptoms in the general population,^18,19,20,21^ making dietary fiber a promising lifestyle factor to investigate in relation to bowel-related symptoms in CRC survivors.

In this study, we evaluated the prevalence of self-reported bowel-related symptoms at 6 months, 2 years, and 5 years after diagnosis in a large prospective cohort of CRC survivors. Stratification based on type of treatment, including surgery type, was performed to investigate symptoms across treatment modalities. Additionally, we investigated whether fiber intake was associated with bowel-related symptoms. Associations between bowel-related symptoms and health-related quality of life (HRQOL) were explored to highlight the potential relevance for clinical practice.

## Methods

### Patients

This cohort study used data from the Colorectal Cancer: Longitudinal, Observational Study on Nutritional and Lifestyle Factors (COLON) study,^22^ a prospective cohort study among CRC survivors providing detailed data on dietary intake and other lifestyle factors as well as clinical outcomes (more details are in the eMethods in Supplement 1). We included all CRC survivors with stage I-IV disease recruited at diagnosis from 11 hospitals in the Netherlands between August 2010 and February 2020 who underwent surgery and excluded those who did not undergo surgery, had missing data on surgery status, or had missing data on bowel-related symptoms to result in the final study population of survivors (eFigure 1 in Supplement 1). The COLON study was approved by the Committee on Research involving Human Subjects, region Arnhem-Nijmegen, the Netherlands. All participants provided written informed consent. Because this study examined exposures and outcomes based on the data of the COLON study in line with the aims that were formulated in the protocol approved by the medical-ethical committee and the informed consent signed by participants, no further approval of this study was necessary. The study followed the Strengthening the Reporting of Observational Studies in Epidemiology (STROBE) reporting guidelines.

### Bowel-Related Symptoms and HRQOL

Data on bowel-related symptoms were obtained via a questionnaire that was provided at 6 months, 2 years, and 5 years after diagnosis. In this questionnaire, the following question was included: “Do you experience any symptoms as a result of your treatment?” Participants could indicate (yes or no) whether they experienced diarrhea, constipation, flatulence or bloating, frequent stools, mucus in stools, and/or a false sense of urgency to defecate (hereafter shortened to *false urgency*) resulting from their CRC treatment. We defined having any bowel-related symptoms as the report of at least 1 of these symptoms. HRQOL at diagnosis and all follow-up time points was assessed using the validated European Organisation for Research and Treatment of Cancer Quality of Life Questionnaire Core 30 (EORTC QLQ-C30),^23^ which is described in more detail in the eMethods in Supplement 1.

### Clinical Data and Sociodemographic and Lifestyle Factors

Hospital records and linkage with the Dutch ColoRectal Audit^24^ and Netherlands Cancer Registry were used to obtain clinical data, including treatment strategy (surgery alone or surgery combined with chemotherapy, radiotherapy, or both) and resection type (hemicolectomy, sigmoid resection, low anterior resection, or abdominoperineal resection). Data on sociodemographic and lifestyle factors were obtained via questionnaires completed at diagnosis and 6 months, 2 years, and 5 years after diagnosis. At these time points, habitual dietary fiber intake was assessed using a 204-item food-frequency questionnaire.^25,26^ Details are included in the eMethods in Supplement 1.

### Statistical Analysis

Characteristics of participants are presented as medians and IQRs or numbers and percentages. To investigate whether fiber intake (per 10-g/d increment) was associated with bowel-related symptoms, we performed multivariable logistic regression analyses to calculate odds ratios (ORs) and 95% CIs. Multivariable models were constructed for each time point (6 months, 2 years, and 5 years after diagnosis) with adjustment for relevant confounders as described in the eMethods in Supplement 1.

Associations between dietary fiber intake and moderate-to-severe diarrhea and constipation were further investigated using the validated EORTC QLQ-C30. Multivariable logistic regression analyses were performed to investigate associations between fiber intake (per 10-g/d increment) and prevalence of moderate-to-severe diarrhea or constipation, defined as a questionnaire response of yes (indicated by a self-report of “quite a bit” or “very much”) vs no, for each time point (at diagnosis and 6 months, 2 years, and 5 years after diagnosis). ORs and 95% CIs were calculated with adjustment for relevant confounders in the same way as described earlier. We also ran models further adjusted for occurrence of moderate-to-severe diarrhea or constipation at diagnosis. Additionally, complete-case analyses were performed, including only participants with data on bowel-related symptoms at all time points.

Associations between bowel-related symptoms (categorical [yes or no]) and HRQOL (continuous) were examined using multivariable linear regression. Regression coefficients (B) and 95% CIs were calculated for each time point (6 months, 2 years, and 5 years after diagnosis). Analyses were adjusted for sex, age at diagnosis, tumor location, and current stoma. Analyses for specific bowel-related symptoms were mutually adjusted for prevalence of the other symptoms.

Analyses were performed between April 2024 and March 2025 using R, version 4.2.1 (R Project for Statistical Computing). GraphPad Prism, version 9.4.1 (GraphPad Software), was used for visualization purposes. ORs with 95% CIs not containing 1 or B coefficients with 95% CIs not containing 0 were considered statistically significant.

## Results

**Figure 1.**
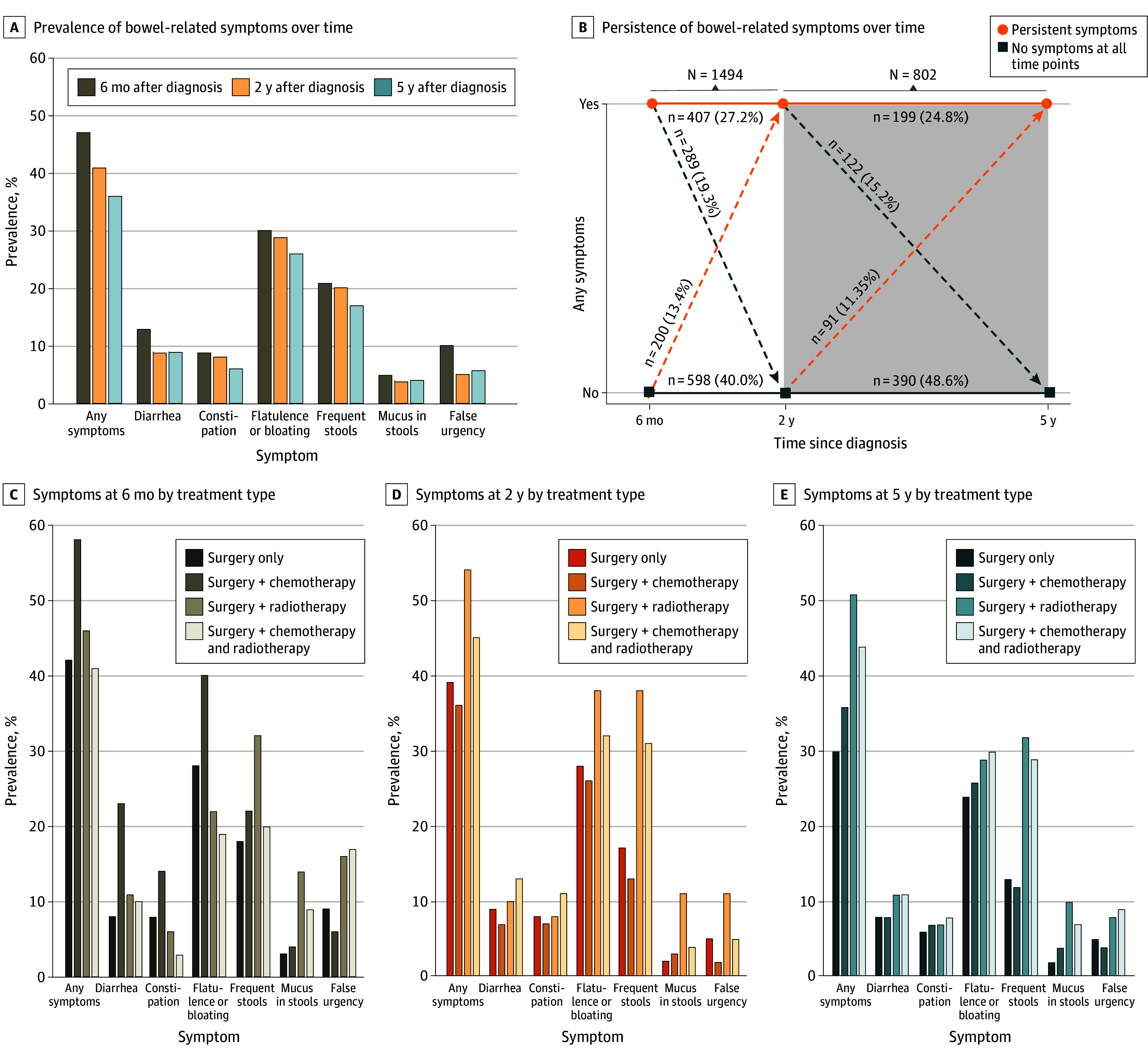
Prevalence and Persistence of Bowel-Related Symptoms Overall and by Type of Neoadjuvant and Adjuvant Treatment at 6 Months, 2 Years, and 5 Years After Diagnosis Among Colorectal Cancer Survivors

Of 2113 CRC survivors in the COLON study, we excluded 26 who did not undergo surgery and 17 who had missing data on surgery status. Of the remaining 2070 participants who underwent surgery, 2038 (98.5%) were alive at 6 months, 1921 (92.8%) at 2 years, and 1719 (83.0%) at 5 years after diagnosis (eFigure 1 in Supplement 1). Reflecting the percentage of questionnaires returned from participants still alive at each time point, data on bowel-related symptoms were available for 1751 (85.9%) of those alive at 6 months and 1511 (78.7%) of those alive at 2 years. At the start of data analyses, 226 of the 1719 participants surviving to 5 years (13.1%) had not yet reached the 5-year time point; thus, of the remaining 1493 (86.9%), data were available for 812 (54.4%) at 5 years after diagnosis (eFigure 1 in Supplement 1). Median age at diagnosis was 66 years (IQR, 61-71 years); of the 1751 survivors with bowel-related symptom data at 6 months, 1115 (63.7%) were men and 636 (36.3%) were women, and 1174 (68.2%) of 1721 with available data on tumor location were diagnosed with colon cancer. Of the surviving participants with available symptom data at each time point, bowel-related symptoms were reported by 817 (46.7%) at 6 months, 614 (40.6%) at 2 years, and 290 (35.7%) at 5 years after diagnosis (Figure 1A). Characteristics of survivors with and without bowel-related symptoms are reported in Table 1. Survivors with a current stoma reported bowel-related symptoms less often at all time points compared with those without a stoma (eTable 1 in Supplement 1).

**Table 1.** Sociodemographic, Clinical, and Lifestyle Characteristics of CRC Survivors Reporting No or Any Bowel-Related Symptoms

Characteristic	Bowel-related symptoms^a^					
	6 mo After diagnosis (n = 1751)		2 y After diagnosis (n = 1511)		5 y After diagnosis (n = 812)	
	None (n = 934 [53.3%])	Any (n = 817 [46.7%])	None (n = 897 [59.4%])	Any (n = 614 [40.6%])	None (n = 522 [64.3%])	Any (n = 290 [35.7%])
Age at CRC diagnosis, median (IQR), y	67 (61-72)	65 (60-71)	67 (61-72)	65 (60-71)	67 (62-72)	65 (60-70)
Sex						
Female	312 (33.4)	324 (39.7)	298 (33.2)	250 (40.7)	182 (34.7)	108 (37.2)
Male	622 (66.6)	493 (60.3)	599 (66.8)	364 (59.3)	340 (65.1)	182 (62.8)
Tumor location^b^						
Colon	625 (68.2)	549 (68.3)	635 (72.2)	375 (62.0)	376 (72.0)	171 (59.2)
Rectum	292 (31.8)	255 (31.7)	245 (27.8)	230 (38.0)	146 (28.0)	121 (40.8)
Cancer stage^c^						
I	258 (28.5)	182 (22.8)	232 (26.7)	166 (27.8)	149 (28.9)	73 (25.7)
II	249 (27.5)	205 (25.7)	245 (28.2)	158 (26.4)	162 (31.4)	69 (24.3)
III	333 (36.8)	364 (45.7)	360 (41.4)	243 (40.6)	188 (36.4)	132 (46.5)
IV	64 (7.1)	46 (5.8)	32 (3.7)	31 (5.2)	17 (3.3)	10 (3.5)
Type of neoadjuvant and adjuvant treatment^d^						
Surgery only	519 (56.7)	379 (47.3)	482 (54.9)	313 (51.9)	296 (56.8)	129 (44.6)
Surgery plus chemotherapy	191 (20.3)	260 (32.4)	233 (26.5)	130 (21.6)	123 (23.6)	68 (23.5)
Surgery plus radiotherapy	98 (10.7)	85 (10.6)	74 (8.4)	86 (14.3)	45 (8.6)	47 (16.3)
Surgery plus chemotherapy and radiotherapy	113 (12.3)	78 (9.7)	89 (10.4)	74 (12.3)	57 (10.9)	45 (15.6)
Type of resection^e^						
Hemicolectomy	358 (38.8)	265 (32.7)	350 (39.3)	187 (30.7)	198 (38.0)	82 (28.3)
Sigmoid resection	191 (20.7)	216 (26.7)	224 (25.2)	131 (21.5)	138 (26.5)	60 (20.7)
Low anterior resection	242 (26.2)	280 (34.6)	201 (22.6)	251 (41.2)	118 (22.6)	129 (44.5)
Abdominoperineal resection	105 (11.4)	25 (3.0)	91 (10.2)	21 (3.4)	52 (10.0)	13 (4.5)
Other^f^	26 (2.8)	24 (3.0)	24 (2.7)	19 (3.1)	15 (2.9)	6 (2.1)
Current stoma^g^	281 (30.1)	49 (12.1)	179 (20.0)	51 (8.3)	114 (21.8)	24 (8.3)
Moderate-to-severe diarrhea at CRC diagnosis^h^	25 (2.7)	95 (11.8)	13 (1.5)	63 (10.5)	6 (1.2)	28 (10.1)
Moderate-to-severe constipation at CRC diagnosis^i^	11 (1.2)	53 (6.6)	9 (1.0)	38 (6.4)	9 (1.8)	13 (4.6)
BMI, median (IQR)^j^	26.0 (23.8-28.4)	26.0 (24.0-28.7)	26.6 (24.5-29.4)	26.3 (24.2-29.0)	26.2 (24.3-29.1)	26.7 (24.1-28.9)
Physical activity, median (IQR), h/wk^k^	8.5 (4.2-15.0)	8.0 (4.4-14.0)	9.5 (5.0-17.5)	10.9 (6.0-18.0)	9.5 (4.2-17.2)	10.0 (5.3-17.9)
Smoking^l^						
Never	301 (32.5)	254 (31.3)	302 (34.0)	175 (28.8)	168 (32.7)	77 (26.7)
Former	562 (60.8)	506 (62.3)	528 (59.5)	383 (63.1)	320 (62.3)	186 (64.6)
Current	62 (6.7)	52 (6.4)	60 (6.5)	49 (8.1)	26 (5.1)	25 (8.7)
Dietary fiber intake, median (IQR), g/d^m^	18.8 (15.3-23.0)	20.1 (16.0-24.1)	18.5 (14.7-23.4)	19.7 (15.6-24.4)	18.2 (15.0-22.4)	19.2 (15.4-22.5)
Energy intake, median (IQR), kcal/d^m^	1765 (1465-2090)	1794 (1484-2153)	1666 (1365-2034)	1770 (1461-2086)	1638 (1345-1959)	1675 (1417-2015)

Abbreviations: BMI, body mass index (calculated as weight in kilograms divided by height in meters squared); CRC, colorectal cancer.

^a^ Data are presented as number (percentage) of survivors unless otherwise indicated.

^b^ Data were missing for 30 survivors at 6 months, 26 at 2 years, and 1 at 5 years.

^c^ Data were missing for 50 survivors at 6 months, 44 at 2 years, and 12 at 5 years.

^d^ Data were missing for 33 survivors at 6 months, 30 at 2 years, and 2 at 5 years.

^e^ Data were missing for 19 survivors at 6 months, 12 at 2 years, and 1 at 5 years.

^f^ Other types of resection include transversectomy (n = 19), subtotal colectomy (n = 12), transanal endoscopic microsurgery (n = 16), and other (n = 3); numbers presented in the table refer to the study population at 6 months after diagnosis.

^g^ Self-reported at corresponding time points after diagnosis; data were missing for 4 survivors at 2 years and 2 at 5 years.

^h^ Defined as experience of “quite a bit” or “very much” moderate-to-severe diarrhea based on the European Organisation for Research and Treatment of Cancer Quality of Life Questionnaire Core 30 at CRC diagnosis. Data were missing for 32 survivors at 6 months, 28 at 2 years, and 39 at 5 years.

^i^ Defined as experience of “quite a bit’” or “very much” moderate-to-severe constipation based on the European Organisation for Research and Treatment of Cancer Quality of Life Questionnaire Core 30 at CRC diagnosis. Data were missing for 28 survivors at 6 months, 35 at 2 years, and 21 at 5 years.

^j^ Data were missing for 10 survivors at 6 months, 9 at 2 years, and 2 at 5 years.

^k^ Refers to moderate-to-vigorous physical activity; data were missing for 8 survivors at 6 months, 29 at 2 years, and 79 at 5 years.

^l^ Data were missing for 14 survivors at 6 months, 17 at 2 years, and 10 at 5 years.

^m^ Data were missing for 27 survivors at 6 months, 33 at 2 years, and 19 at 5 years.

Persistence of bowel-related symptoms over time is shown in Figure 1B. Of the participants with available symptom data, approximately a quarter consistently reported bowel-related symptoms between 6 months and 2 years (407 of 1494 [27.2%]) and between 2 years and 5 years (199 of 802 [24.8%]) after diagnosis. A substantial number of participants developed symptoms over time (200 of 1494 [13.4%] after 6 months and 91 of 802 [11.3%] after 2 years after diagnosis).

### Bowel-Related Symptoms and Treatment Strategy

When evaluating bowel-related symptoms by treatment strategy, divergent patterns were found for CRC survivors who underwent (1) surgery only, (2) surgery and chemotherapy, (3) surgery and radiotherapy, and (4) surgery and a combination of chemotherapy and radiotherapy (Figure 1C-E). The prevalence of bowel-related symptoms at 6 months after diagnosis was highest in the survivors who had received chemotherapy or were still receiving chemotherapy (260 of 446 [58.3%]). At 6 months after diagnosis, 166 of 430 participants receiving surgery and chemotherapy (38.6%) had completed their chemotherapy. Prevalence of bowel-related symptoms was 169 of 264 (64.0%) in those still undergoing chemotherapy and 83 of 166 (50.0%) in those who completed chemotherapy. In contrast, bowel-related symptoms at 2 years and 5 years after diagnosis were most common in survivors who received radiotherapy (86 of 160 [53.8%] at 2 years and 47 of 92 [51.1%] at 5 years). Prevalence of bowel-related symptoms in those who received radiotherapy increased between 6 months and 2 years and remained stable between 2 years and 5 years after diagnosis, suggesting that these symptoms may be late-onset adverse effects of treatment (Figure 1C-E).

### Bowel-Related Symptoms and Resection Type

To verify whether bowel-related symptoms were more common in survivors who underwent low anterior resection compared with other surgical procedures, we presented symptoms according to surgical procedure (hemicolectomy, sigmoid resection, low anterior resection, and abdominoperineal resection) and type of neoadjuvant and adjuvant treatment (eTable 2 in Supplement 1). When comparing survivors exclusively receiving hemicolectomy with survivors exclusively receiving low anterior resection, the prevalence of bowel-related symptoms was higher in the latter group at 6 months (142 of 396 [35.9%] vs 104 of 197 [52.8%]), 2 years (131 of 350 [37.4%] vs 88 of 176 [50.0%]), and 5 years (52 of 184 [28.3%] vs 40 of 91 [44.0%]) after diagnosis. Prevalence of bowel-related symptoms at 2 years after diagnosis was higher among survivors who also received radiotherapy (77 of 126 [61.1%]) or chemotherapy and radiotherapy (59 of 99 [59.6%]) compared with survivors exclusively receiving low anterior resection (88 of 176 [50.0%]). This pattern remained consistent at 5 years after diagnosis in those who had received radiotherapy (40 of 72 [55.6%]) and those who had received both chemotherapy and radiotherapy (40 of 63 [63.5%]) vs those who received a low anterior resection only (40 of 91 [44.0%]). Prevalence of bowel-related symptoms after a sigmoid resection was comparable with that after a hemicolectomy, whereas symptoms were less common after an abdominoperineal resection compared with all other resections (eTable 2 in Supplement 1). Complete-case analyses with participants who completed questionnaires at all time points (795 [38.4%] after excluding 2 patients with missing data on treatment type) resulted in comparable findings (eTable 3 in Supplement 1).

### Dietary Fiber Intake and Bowel-Related Symptoms

To study associations between dietary fiber intake and bowel-related symptoms, multivariable logistic regression analyses were performed with adjustment for relevant sociodemographic, clinical, and other lifestyle factors (Table 2). A higher fiber intake (per 10-g/d increment) was associated with a lower prevalence of diarrhea at 2 years (OR, 0.48; 95% CI, 0.31-0.73) and 5 years (OR, 0.48; 95% CI, 0.26-0.90) after diagnosis as well as a lower prevalence of mucus in stools at 6 months (OR, 0.61; 95% CI, 0.37-0.99) and 2 years (OR, 0.52; 95% CI, 0.27-0.99) after diagnosis. In contrast, a higher fiber intake was associated with a higher prevalence of flatulence or bloating at 6 months after diagnosis (OR, 1.57; 95% CI, 1.24-2.01). No associations were found between fiber intake and other bowel-related symptoms.

**Table 2.** Associations Between Dietary Fiber Intake and Prevalence of Bowel-Related Symptoms in Colorectal Cancer Survivors^a^

Time after diagnosis	Dietary fiber intake, median (IQR), g/d	OR (95% CI)					
		Diarrhea	Constipation^b^	Flatulence or bloating	Frequent stools^c^	Mucus in stools^d^	False urgency^e^
6 mo	19.4 (15.6-23.6)	0.83 (0.59-1.15)	1.01 (0.67-1.51)	1.57 (1.24-2.01)	1.09 (0.81-1.45)	0.61 (0.37-0.99)	0.97 (0.65-1.43)
2 y	19.0 (15.0-23.7)	0.48 (0.31-0.73)	1.06 (0.68-1.63)	1.13 (0.87-1.46)	0.97 (0.71-1.32)	0.52 (0.27-0.99)	1.51 (0.87-2.61)
5 y	18.5 (15.0-22.4)	0.48 (0.26-0.90)	1.02 (0.51-1.98)	1.28 (0.88-1.85)	1.23 (0.78-1.93)	0.49 (0.19-1.24)	0.80 (0.36-1.69)

Abbreviation: OR, odds ratio.

^a^ All multivariable logistic regression models were adjusted for sex, age at diagnosis (continuous in years), tumor location (colon, rectum), and energy intake (continuous in kcal/d).

^b^ Analyses were also adjusted for smoking status (current, former, or never).

^c^ Analyses were also adjusted for current stoma (yes or no).

^d^ Analyses were also adjusted for current stoma (yes or no) and level of moderate-to-vigorous physical activity (continuous, in hours/week).

^e^ Analyses were also adjusted for smoking status (current, former, or never), current stoma (yes or no), and level of moderate-to-vigorous physical activity (continuous, in hours/week).

### Dietary Fiber Intake and Moderate-to-Severe Diarrhea and Constipation

Associations between dietary fiber intake and diarrhea as well as constipation were further investigated using the validated EORTC QLQ-C30. Based on this questionnaire, we could evaluate severity of diarrhea or constipation as well as adjust for the occurrence of diarrhea or constipation at time of diagnosis (Table 3). Higher fiber intake (per 10-g/d increment) was associated with a lower prevalence of moderate-to-severe diarrhea at 6 months (OR, 0.44; 95% CI, 0.28-0.70) and 2 years (OR, 0.53; 95% CI, 0.30-0.94) after diagnosis, while the association was not statistically significant at 5 years (OR, 0.43; 95% CI, 0.16-1.13) after diagnosis—all independent of occurrence of moderate-to-severe diarrhea at time of diagnosis. No associations were found between fiber intake and moderate-to-severe constipation (eTable 4 in Supplement 1). Complete-case analyses resulted in comparable findings (eTables 5 and 6 in Supplement 1).

**Table 3.** Associations Between Dietary Fiber Intake and Prevalence of Moderate-to-Severe Diarrhea, Based on Data From the EORTC QLQ-C30 Questionnaire, in Colorectal Cancer Survivors^a^

Time	Dietary fiber intake, median (IQR), g/d	Moderate-to-severe diarrhea^b^		Moderate-to-severe diarrhea, adjusted for occurrence of diarrhea at diagnosis^b^^,^^c^	
		No. of events/total population (%)	OR (95% CI)	No. of events/total population (%)	OR (95% CI)
At diagnosis	19.7 (15.8-24.2)	203/1828 (11.1)	0.54 (0.38-0.76)	NA	NA
At 6 mo after diagnosis	19.4 (15.6-23.6)	117/1671 (7.0)	0.45 (0.28-0.71)	116/1642 (7.1)	0.44 (0.28-0.70)
At 2 y after diagnosis^d^	19.0 (15.0-23.7)	72/1419 (5.1)	0.53 (0.30-0.93)	71/1397 (5.1)	0.53 (0.30-0.94)
At 5 y after diagnosis^e^	18.5 (15.0-22.4)	29/744 (3.9)	0.43 (0.16-1.13)	29/735 (3.9)	0.43 (0.16-1.13)

Abbreviations: EORTC QLQ-C30, European Organisation for Research and Treatment of Cancer Quality of Life Questionnaire Core 30; NA, not applicable; OR, odds ratio.

^a^ All models were adjusted for sex, age at diagnosis (continuous in years), tumor location (colon, rectum), and energy intake (continuous in kcal/d) at all time points.

^b^ Defined as experience of “quite a bit” or “very much” moderate-to-severe diarrhea.

^c^ All models were further adjusted for occurrence of moderate-to-severe diarrhea at diagnosis (yes or no) based on data from the EORTC QLQ-C30.

^d^ Model was further adjusted for level of moderate-to-vigorous physical activity (continuous in hours/week).

^e^ Model was further adjusted for cancer stage (I, II, III, or IV), current stoma (yes or no), body mass index (calculated as weight in kilograms divided by height in meters squared) (continuous), and smoking status (current, former, or never).

### Bowel-Related Symptoms and HRQOL

**Figure 2.**
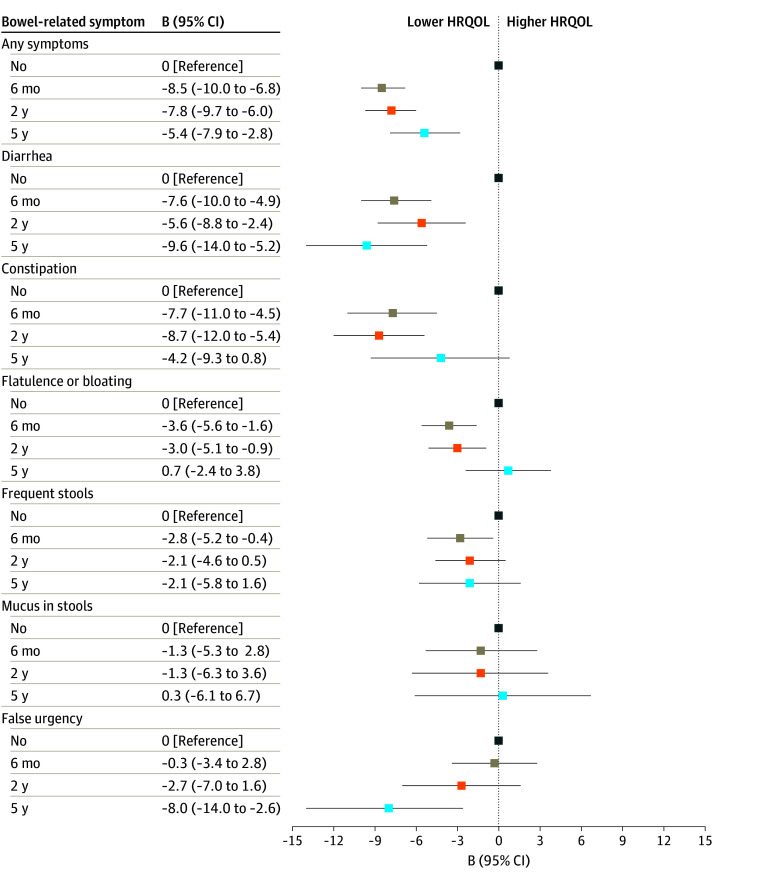
Associations Between Bowel-Related Symptoms and Health-Related Quality of Life (HRQOL) HRQOL was based on European Organisation for Research and Treatment of Cancer Quality of Life Questionnaire Core 30 (EORTC QLQ-C30) global health status and quality of life scores at 6 months, 2 years, and 5 years after diagnosis. A total of 1698 survivors were included in the analyses at 6 months, 1452 at 2 years, and 796 at 5 years after diagnosis. Linear regression models were adjusted for sex, age at diagnosis (continuous in years), tumor location (colon, rectum), and current stoma (yes or no). Analyses for specific bowel-related symptoms were mutually adjusted for prevalence of the other bowel-related symptoms.

Based on our findings of fiber intake being associated, albeit in different directions, with diarrhea, mucus in stools, and flatulence or bloating, we aimed to explore which bowel-related symptoms were associated with HRQOL. Differences in HRQOL scores for survivors with and without these bowel-related symptoms are shown in eFigure 2 in Supplement 1. Multivariable logistic regression analyses with adjustment for age at diagnosis, sex, tumor location, and current stoma showed that having any bowel-related symptoms (vs no symptoms) was associated with a lower HRQOL score at all time points (Figure 2). Across the specific symptoms, diarrhea was associated with lower HRQOL scores at all time points and with the lowest HRQOL score out of all symptoms (B, −9.6 [95% CI, −14.0 to −5.2] at 5 years after diagnosis) (Figure 2). Mucus in stools was not associated with HRQOL, and flatulence or bloating was only associated with a lower HRQOL at 6 months and 2 years after diagnosis (Figure 2).

## Discussion

We described the prevalence of bowel-related symptoms up to 5 years after diagnosis in CRC survivors participating in a large prospective cohort study. Almost half of the survivors (46.7%) reported bowel-related symptoms at 6 months, whereas more than one-third (35.7%) still experienced bowel-related symptoms at 5 years after diagnosis. Symptoms at 6 months were mainly reported by survivors who received chemotherapy, while symptoms at 2 years and 5 years after diagnosis were most prevalent in those who received radiotherapy. A higher habitual dietary fiber intake was associated with a lower prevalence of diarrhea at 6 months and 2 years after diagnosis, while at 5 years after diagnosis the association had a comparable OR but was not statistically significant, likely due to the lower sample size. Of the studied bowel-related symptoms, only diarrhea was associated with lower HRQOL scores at all time points. Additionally, a higher fiber intake was associated with a lower prevalence of mucus in stools at 6 months and 2 years after diagnosis, but mucus in stools was not associated with HRQOL. A higher fiber intake was also associated with a higher prevalence of flatulence or bloating at 6 months but not at 2 years and 5 years after diagnosis.

In clinical practice, persistent bowel-related symptoms in CRC survivors have been primarily attributed to low anterior resections.^5,7^ Our findings indeed showed a higher prevalence of bowel-related symptoms in survivors who underwent a low anterior resection compared with those with a hemicolectomy or sigmoid resection. However, prevalence of bowel-related symptoms was highest in survivors who had received radiotherapy in combination with a low anterior resection. Other studies also showed that in addition to surgery, radiotherapy is an important risk factor of persistent bowel-related symptoms in rectal cancer survivors.^27,28,29,30^ Radiotherapy may cause chronic intestinal inflammation or ischemia and fibrosis leading to bowel adhesions, potentially contributing to impaired bowel function and symptoms.^31,32,33^ Although health care professionals indicate that many CRC survivors fear having a permanent colostomy,^34,35,36^ our data showed that survivors with a current stoma had fewer bowel-related symptoms compared with those without a stoma. These findings may inform clinicians in weighing advantages and disadvantages of restorative resections and fuel well-informed shared decision-making.

In our study, diarrhea was associated with lower HRQOL scores at all time points. Many other studies have shown a higher prevalence of diarrhea (measured with EORTC QLQ-C30) in CRC survivors compared with age- and sex-matched population controls.^37,38,39,40^ A case-control study of 1262 CRC survivors found a higher prevalence of diarrhea, regardless of time since diagnosis (range, 5-16 years), compared with 1689 population controls.^40^ In the same study, diarrhea was associated with a lower quality of life.^40^ Diarrhea is also frequently reported by cancer survivors who received radiotherapy.^31,41^ We observed that a higher dietary fiber intake was associated with a lower prevalence of diarrhea in the years after diagnosis. These findings were consistent when using data of the validated EORTC QLQ-C30, only considering moderate-to-severe diarrhea, which could make our findings meaningful for clinical practice.

Literature on fiber intake and diarrhea in CRC survivors is scarce.^10^ Contrasting our findings, Kenkhuis et al^42^ did not find an association between fiber intake and severity of diarrhea up to 2 years after treatment in CRC survivors, although the estimate (β, −2.1; 95% CI, −5.1 to 0.9) pointed in the same direction as our findings. Also, the sample size of their study was comparatively modest (n = 396), which may have limited statistical power to detect associations. Increasing fiber intake has been proposed as a potential strategy to reduce adverse effects of pelvic radiotherapy in cancer patients, but evidence is still limited and inconclusive.^43,44,45^

Likely, the role of dietary fiber in maintaining gut health could explain inverse associations between fiber intake and diarrhea. Maintaining or restoring gut health is especially relevant in CRC survivors, as their gut health is challenged by surgical procedures and potential neoadjuvant and adjuvant treatments.^46,47,48,49,50,51,52^ Dietary fiber impacts the gut microbiota,^21,53^ with potential relevance for restoring microbial disturbances caused by cancer treatment.^46,47,48,49,50,51,52^ Dietary fiber, with its water-holding and bulking properties, also contributes to improved stool patterns.^21,54,55,56^ It should be noted that besides potential protective associations with diarrhea and mucus in stools, we also found that a higher fiber intake was associated with higher prevalence of flatulence or bloating at 6 months but not at 2 and 5 years after diagnosis. Compared with diarrhea, flatulence or bloating was associated with a lesser reduction in HRQOL in this study. Flatulence or bloating are often observed after an increased fiber intake but are strongly dependent on fiber source.^54,57,58^ Therefore, the most effective as well as tolerable fiber sources need to be identified for future studies aiming at symptom management in oncologic populations.

### Strengths and Limitations

Strengths of this study include the repeated data on bowel-related symptoms until 5 years after diagnosis, which allowed investigation over time. Furthermore, we compared prevalence and determinants of bowel-related symptoms across various treatments, while earlier studies were predominantly restricted to patient groups receiving particular treatments such as low anterior resection. Moreover, this is one of the first studies investigating dietary fiber intake in relation to bowel-related symptoms in CRC survivors with adjustment for clinical and other lifestyle factors.

This study also has limitations. One is that residual confounding inherent to the observational design cannot be ruled out, even though we included relevant sociodemographic, clinical, and lifestyle factors in the analyses. Also, reverse causation cannot be entirely ruled out. Participants could have lowered their fiber intake when they experienced diarrhea, although earlier work demonstrated that CRC survivors reported increasing their fiber intake in management of bowel-related symptoms.^15,16,17^

Since assessment of bowel-related symptoms was timed relative to date of diagnosis rather than completion of treatment, survivors who underwent neoadjuvant treatment may have completed their questionnaires sooner after surgery as compared with survivors with other treatment strategies. Prevalences of bowel-related symptoms at 6 months after diagnosis were, however, comparable for survivors with and without neoadjuvant treatment. At 2 years and 5 years after diagnosis, when differences in time since surgery became less relevant due to longer follow-up time, prevalences of bowel-related symptoms were higher in survivors who received neoadjuvant treatment (vs those who did not), indicating that neoadjuvant treatment, rather than time since surgery, might be linked to these symptoms.

We had no data on treatment modalities provided for management of bowel-related symptoms, such as pelvic floor physiotherapy or loperamide use. Additionally, we had no data on bowel-related symptoms at diagnosis (except for diarrhea and constipation) nor a matched comparison group of individuals without cancer to assess whether the prevalence of symptoms was increased compared with the general population.^59^ It should be noted, however, that we asked specifically for bowel-related symptoms related to CRC treatment. Also, survivors with inflammatory bowel diseases, known to have prominent bowel-related symptoms, were not eligible for this study, further implying that the reported symptoms were specifically associated with CRC or its treatment.

## Conclusions

In this cohort study among 1751 CRC survivors, we found that the prevalence of bowel-related symptoms was considerably high in CRC survivors up to 5 years after diagnosis, especially in those who had undergone radiotherapy in combination with low anterior resection. As these symptoms severely impair quality of life,^55,56,60,61^ CRC survivors with bowel-related symptoms may benefit from management of those symptoms as part of their cancer care. Higher intake of dietary fiber was associated with a lower prevalence of diarrhea, which implies the potential role of nutritional counseling in management of bowel-related symptoms. Our findings provide an evidence base for investigating nutritional strategies, including increasing dietary fiber intake, to target bowel-related symptoms in CRC survivors.

## Notes

Supplement 1. **eMethods.** COLON Study
eReferences
**eFigure 1.** Flow Diagram of Colorectal Cancer Survivors at the 3 Respective Time Points, Namely 6 Months, 2 Years, and 5 Years After Diagnosis **eFigure 2.** Mean Scores for Health-Related Quality of Life (HRQOL) and Functioning Subscales Among Colorectal Cancer Survivors **eTable 1.** Prevalence of Bowel-Related Complaints in Colorectal Cancer Survivors With and Without a Stoma **eTable 2.** Prevalence of Bowel-Related Complaints in Colorectal Cancer Survivors Presented by Type of Surgery and (Neo)adjuvant Treatments **eTable 3.** Prevalence of Bowel-Related Complaints in Colorectal Cancer Survivors Presented by Type of Surgery and (Neo)adjuvant Treatments—Complete-Case Analysis Across 795 Survivors **eTable 4.** Associations Between Dietary Fiber Intake and Prevalence of Moderate-to-Severe Constipation, Based on Data From the EORTC QLQ-C30 Questionnaire, in Colorectal Cancer Survivors **eTable 5.** Associations Between Dietary Fiber Intake and Prevalence of Moderate-to-Severe Diarrhea, Based on Data From the EORTC QLQ-C30 Questionnaire, in Colorectal Cancer Survivors—Complete-Case Analysis Across 690 Survivors **eTable 6.** Associations Between Dietary Fiber Intake and Prevalence of Moderate-to-Severe Constipation, Based on Data From the EORTC QLQ-C30 Questionnaire, in Colorectal Cancer Survivors—Complete-Case Analysis Across 708 Survivors

Supplement 2.
Data Sharing Statement
