# Supplementary material for: Bowel-Related Symptoms and Dietary Fiber Intake in Colorectal Cancer Survivors
Source: JAMA Netw Open. 2025 Nov 10;8(11):e2542147. doi: 10.1001/jamanetworkopen.2025.42147 (PMC12603865; doi:10.1001/jamanetworkopen.2025.42147)
Supplement: Supplement 1. — eMethods. COLON Study eReferences eFigure 1. Flow Diagram of Colorectal Cancer Survivors at the 3 Respective Time Points, Namely 6 Months, 2 Years, and 5 Years After Diagnosis eFigure 2. Mean Scores for Health-Related Quality of Life (HRQOL) and Functioning Subscales Among Colorectal Cancer Survivors eTable 1. Prevalence of Bowel-Related Complaints in Colorectal Cancer Survivors With and Without a Stoma eTable 2. Prevalence of Bowel-Related Complaints in Colorectal Cancer Survivors Presented by Type of Surgery and (Neo)adjuvant Treatments eTable 3. Prevalence of Bowel-Related Complaints in Colorectal Cancer Survivors Presented by Type of Surgery and (Neo)adjuvant Treatments—Complete-Case Analysis Across 795 Survivors eTable 4. Associations Between Dietary Fiber Intake and Prevalence of Moderate-to-Severe Constipation, Based on Data From the EORTC QLQ-C30 Questionnaire, in Colorectal Cancer Survivors eTable 5. Associations Between Dietary Fiber Intake and Prevalence of Moderate-to-Severe Diarrhea, Based on Data From the EORTC QLQ-C30 Questionnaire, in Colorectal Cancer Survivors—Complete-Case Analysis Across 690 Survivors eTable 6. Associations Between Dietary Fiber Intake and Prevalence of Moderate-to-Severe Constipation, Based on Data From the EORTC QLQ-C30 Questionnaire, in Colorectal Cancer Survivors—Complete-Case Analysis Across 708 Survivors [file jamanetwopen-e2542147-s001.pdf]

Supplementary Online Content

Klaassen-Dekker N, Witteman BJM, van Heek NT, et al. Bowel-related symptoms and dietary fiber intake in colorectal cancer survivors. *JAMA Netw Open*. 2025;8(11):e2542147. doi:10.1001/jamanetworkopen.2025.42147

eMethods. COLON Study

eReferences

eFigure 1. Flow Diagram of Colorectal Cancer Survivors at the 3 Respective Time Points, Namely 6 Months, 2 Years, and 5 Years After Diagnosis

eFigure 2. Mean Scores for Health-Related Quality of Life (HRQOL) and Functioning Subscales Among Colorectal Cancer Survivors

eTable 1. Prevalence of Bowel-Related Complaints in Colorectal Cancer Survivors With and Without a Stoma

eTable 2. Prevalence of Bowel-Related Complaints in Colorectal Cancer Survivors Presented by Type of Surgery and (Neo)adjuvant Treatments

eTable 3. Prevalence of Bowel-Related Complaints in Colorectal Cancer Survivors Presented by Type of Surgery and (Neo)adjuvant Treatments—Complete-Case Analysis Across 795 Survivors

eTable 4. Associations Between Dietary Fiber Intake and Prevalence of Moderate-to-Severe Constipation, Based on Data From the EORTC-QLQ-C30 Questionnaire, in Colorectal Cancer Survivors

eTable 5. Associations Between Dietary Fiber Intake and Prevalence of Moderate-to-Severe Diarrhea, Based on Data From the EORTC-QLQ-C30 Questionnaire, in Colorectal Cancer Survivors—Complete-Case Analysis Across 690 Survivors

eTable 6. Associations Between Dietary Fiber Intake and Prevalence of Moderate-to-Severe Constipation, Based on Data From the EORTC-QLQ-C30 Questionnaire, in Colorectal Cancer Survivors—Complete-Case Analysis Across 708 Survivors

This supplementary material has been provided by the authors to give readers additional information about their work.

© 2025 Klaassen-Dekker N et al. *JAMA Network Open*.

eMETHODS

COLON study

The COLON (COlorectal cancer: Longitudinal, Observational study on Nutritional and lifestyle factors) study is a prospective cohort study among CRC survivors (n=2113) providing detailed data on dietary intake and other lifestyle factors, as well as clinical outcomes[1]. Patients newly diagnosed with CRC were recruited from 11 hospitals in the Netherlands and followed during and after treatment. Both men and women, aged ≥18 years, with any stage of disease were included. Non-Dutch speaking patients, or patients with a history of CRC or (partial) bowel resection, hereditary CRC syndromes (e.g., Lynch syndrome, Familial Adenomatous Polyposis, Peutz-Jegher), dementia or mental conditions that hampered completion of the questionnaires, were not included. Also, CRC survivors with inflammatory bowel disease were not eligible for the COLON study.

All participants provided written informed consent. The COLON study was approved by the Committee on Research involving Human Subjects, region Arnhem-Nijmegen, the Netherlands (2009–349), and was registered at clinicaltrials.gov with identifier NCT03191110.

Quality of life

Data on health-related quality of life at diagnosis and 6 months, 2 years, and 5 years after diagnosis were collected using the validated European Organization for Research and Treatment of Cancer Quality of Life Questionnaire C30 (EORTC-QLQ-C30) version 3.0[2]. The EORTC-QLQ-C30 assesses global health status/QoL (hereafter referred to as HRQoL), and functioning subscales (physical, cognitive, role, social, and emotional). Scores ranged from 0-100 after linear transformation performed according to the scoring manual[3], and higher scores represent a better quality of life.

© 2025 Klaassen-Dekker N et al. *JAMA Network Open*.

Additionally, the symptom scales of the EORTC-QLQ-C30 were used to further evaluate the prevalence of self-reported moderate-to-severe diarrhea or moderate-to-severe constipation experienced by the indicated time points, which allowed for the consideration of the severity of reported diarrhea or constipation. In the EORTC-QLQ-C30 questionnaire, participants were asked whether they respectively experienced 'not at all', 'a little', 'quite a bit', or 'very much' diarrhea or constipation during the last week. In our analyses, moderate-to-severe diarrhea or constipation was defined based on the combined self-reports for 'quite a bit' and 'very much' diarrhea or constipation in the past week. As the EORTC-QLQ-C30 was also completed at time of diagnosis, we were also able to consider pre-existing diarrhea or constipation at time of diagnosis.

The prevalence of moderate-to-severe diarrhea was 11.3%, 7.0%, 5.1%, and 4.3% at diagnosis, 6 months, 2 years, and 5 years after diagnosis, respectively. At these timepoints, moderate-to-severe constipation was reported by 8.8%, 3.8%, 3.2%, and 2.4% of the population, respectively. In total, 0.5-1.5% of the population reported both moderate-to-severe diarrhea and constipation simultaneously.

Clinical data

Hospital records and linkage with the Dutch ColoRectal Audit (DCRA)[4] and Netherlands Cancer Registry were used to obtain clinical data including tumor location (colon or rectum), cancer stage (I-IV), type of (neo)adjuvant treatment (surgery only, surgery + chemotherapy, surgery + radiotherapy, or surgery + chemo- and radiotherapy), and type of resection (hemicolectomy, sigmoid resection, low anterior resection, or other resections). Additionally, participants were asked whether they had a current stoma when completing the questionnaires on bowel-related complaints.

© 2025 Klaassen-Dekker N et al. *JAMA Network Open*.

Sociodemographic and lifestyle factors

Data on sociodemographic and lifestyle factors were obtained via questionnaires that were also completed at diagnosis, and at 6 months, 2 years, and 5 years after diagnosis. Body mass index (BMI in kg/m^2) was calculated based on self-reported weight and height. Smoking status was reported as current, former, or never smoker. The validated Short QUestionnaire to ASsess Health-enhancing physical activity (SQUASH) was used to assess the level of moderate-to-vigorous physical activity in hours per week[5]. A 204-item semi-quantitative food frequency questionnaire (FFQ)[6,7] was used to assess habitual dietary intake during the previous month. Total habitual dietary fiber intake (g/day) and energy intake (kcal/day) were calculated based on frequency of intake, number of portions, and standard portion size of relevant food items using data from the Dutch food composition table[8].

Covariates

Multivariable logistic regression analyses were performed to investigate the associations between habitual dietary fiber intake and prevalence of bowel-related complaints with adjustment for age at diagnosis, sex, tumor location, and energy intake[9]. These variables were identified as confounders based on the literature, as it has been consistently shown that bowel-related complaints were more common in CRC survivors with higher age[10,11] and among women[10–12], and that colon and rectal cancer have a distinctive etiology, treatment, and type of associated bowel-related complaints[13–15]. Other potential confounders, including cancer stage, type of resection, type of (neo)adjuvant treatment, current stoma, BMI, level of physical activity, and smoking status were added one by one to the models. When potential confounders changed the risk estimates by more than 10%, these variables were included in the final models.

© 2025 Klaassen-Dekker N et al. *JAMA Network Open*.

eREFERENCES

1. Winkels RM, Heine-Bröring RC, van Zutphen M, et al. The COLON study: Colorectal cancer: Longitudinal, Observational study on Nutritional and lifestyle factors that may influence colorectal tumour recurrence, survival and quality of life. *BMC Cancer*. 2014;14(1):374. doi:10.1186/1471-2407-14-374
2. Fayers P, Bottomley A. Quality of life research within the EORTC—the EORTC QLQ-C30. *Eur J Cancer*. 2002;38(SUPPL. 4):125-133. doi:10.1016/S0959-8049(01)00448-8
3. Fayers P, Aaronson N, Bjordal K, et al. *The EORTC QLQ-C30 Scoring Manual (3rd Edition)*.; 2001.
4. Van Leersum NJ, Snijders HS, Henneman D, et al. The Dutch Surgical Colorectal Audit. *Eur J Surg Oncol*. 2013;39(10):1063-1070. doi:10.1016/J.EJSO.2013.05.008
5. Wendel-Vos GCW, Schuit AJ, Saris WHM, Kromhout D. Reproducibility and relative validity of the short questionnaire to assess health-enhancing physical activity. *J Clin Epidemiol*. 2003;56(12):1163-1169. doi:10.1016/S0895-4356(03)00220-8
6. Feunekes IJ, Van Staveren WA, Graveland F, Vos J De, Burema J. Reproducibility of a semiquantitative food frequency questionnaire to assess the intake of fats and cholesterol in the Netherlands. *Int J Food Sci Nutr*. 1995;46(2):117-123. doi:10.3109/09637489509012539
7. Siebelink E, Geelen A, De Vries JHM. Self-reported energy intake by FFQ compared with actual energy intake to maintain body weight in 516 adults. *Br J Nutr*. 2011;106(2):274-281. doi:10.1017/S0007114511000067
8. National Institute for Public Health. Nederlands voedingsstoffenbestand (NEVO). 2011. https://www.rivm.nl/nederlands-voedingsstoffenbestand
9. Willett WC, Howe GR, Kushi LH. Adjustment for total energy intake in epidemiologic studies. *Am J Clin Nutr*. 1997;65(4):1220S-1228S. doi:10.1093/AJCN/65.4.1220S
10. Garfinkle R, Boutros M. Low Anterior Resection Syndrome: Predisposing Factors and Treatment. *Surg Oncol*. 2022;43(101691). doi:10.1016/J.SURONC.2021.101691
11. Sandberg S, Asplund D, Bisgaard T, et al. Low anterior resection syndrome in a Scandinavian population of patients with rectal cancer: a longitudinal follow-up within the QoLiRECT study. *Colorectal Disease*. 2020;22(10):1367-1378. doi:10.1111/CODI.15095
12. van Heinsbergen M, den Haan N, Maaskant-Braat AJ, et al. Functional bowel complaints and quality of life after surgery for colon cancer: prevalence and predictive factors. *Colorectal Disease*. 2020;22(2):136-145. doi:10.1111/CODI.14818
13. Verkuijl SJ, Jonker JE, Trzpis M, Burgerhof JGM, Broens PMA, Furnée EJB. Functional outcomes of surgery for colon cancer: A systematic review and meta-analysis. *Eur J Surg Oncol*. 2021;47(5):960-969. doi:10.1016/J.EJSO.2020.11.136
14. van der Sijp MPL, Bastiaannet E, Mesker WE, et al. Differences between colon and rectal cancer in complications, short-term survival and recurrences. *Int J Colorectal Dis*. 2016;31(10):1683-1691. doi:10.1007/S00384-016-2633-3
15. Murphy N, Ward HA, Jenab M, et al. Heterogeneity of Colorectal Cancer Risk Factors by Anatomical Subsite in 10 European Countries: A Multinational Cohort Study. *Clinical Gastroenterology and Hepatology*. 2019;17(7):1323-1331.e6. doi:10.1016/J.CGH.2018.07.030

© 2025 Klaassen-Dekker N et al. *JAMA Network Open*.

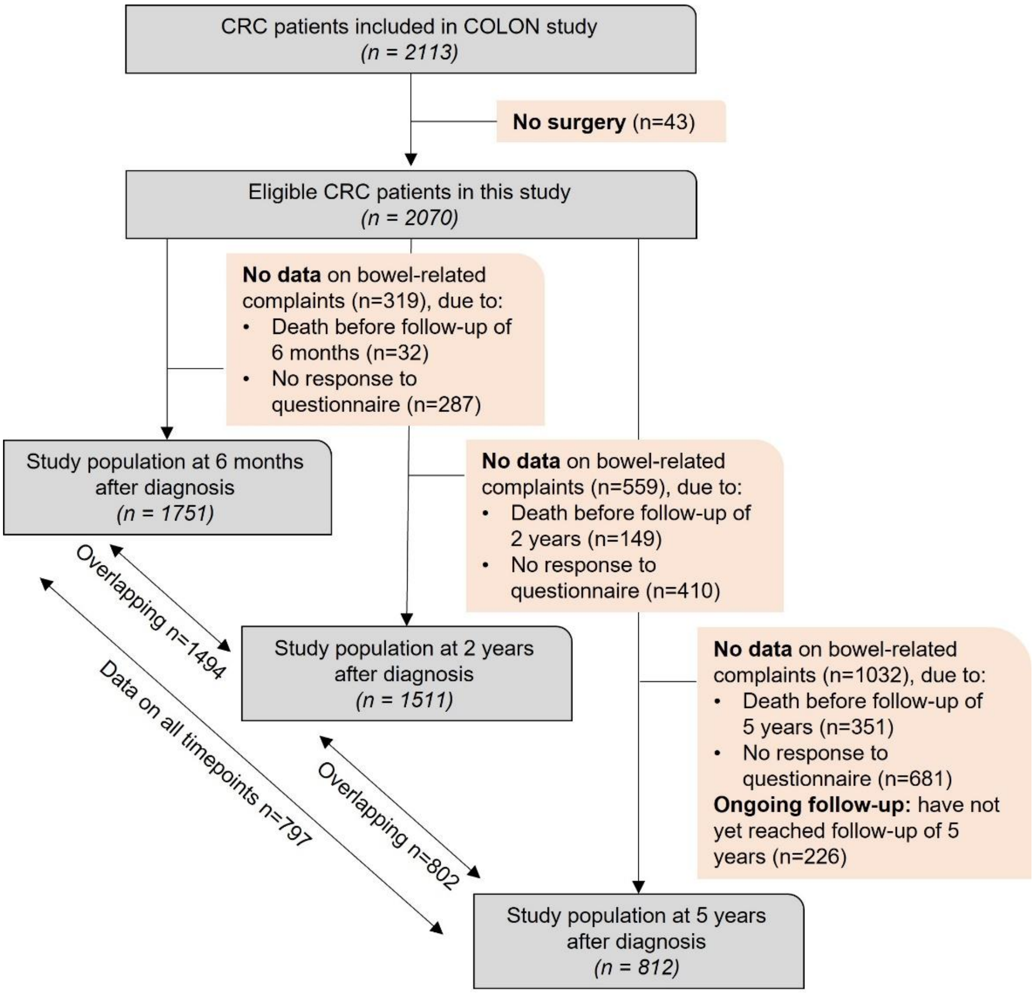

eFigure 1. *Flow diagram of colorectal cancer survivors at the three respective time points, namely 6 months, 2 years, and 5 years after diagnosis.*

© 2025 Klaassen-Dekker N et al. *JAMA Network Open*.

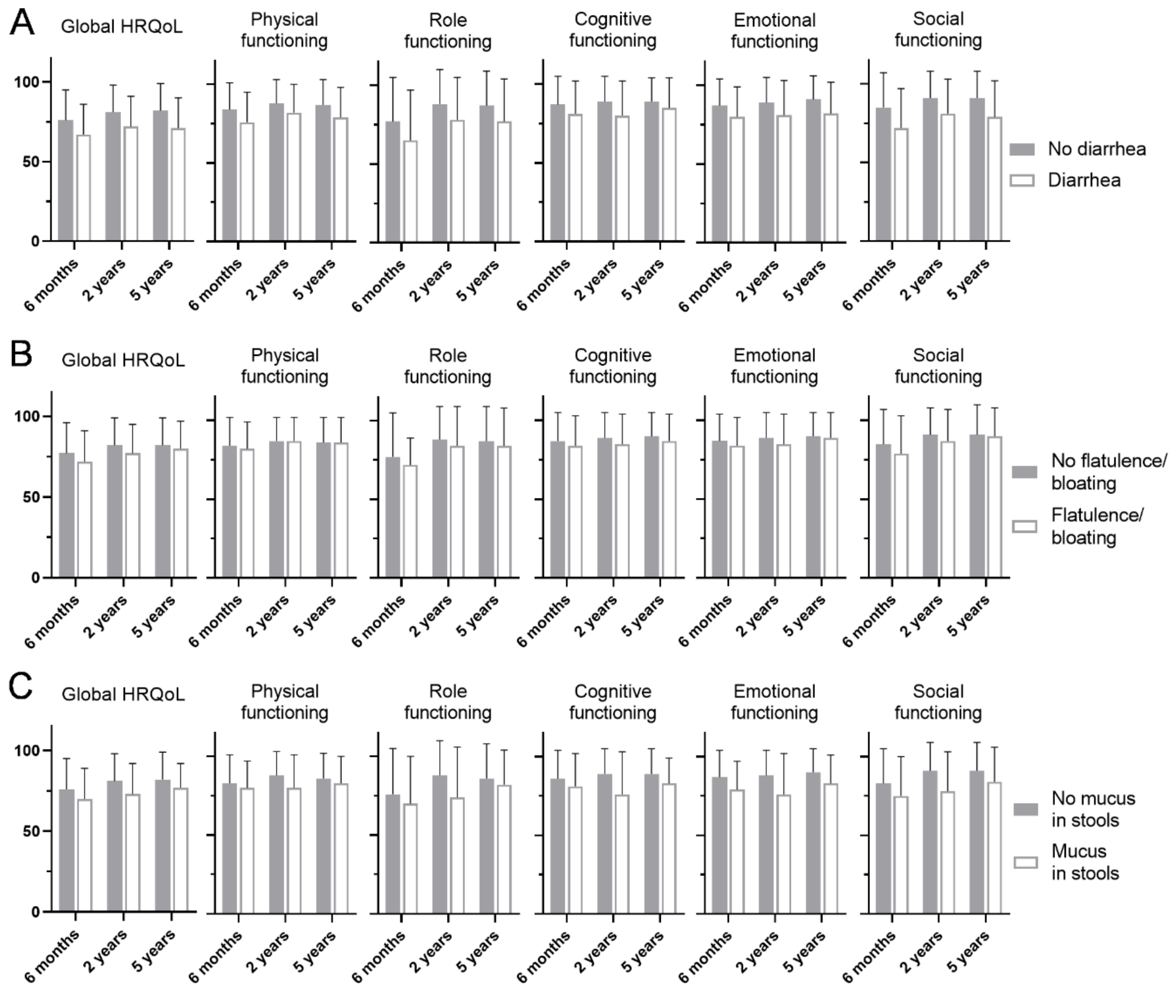

eFigure 2. *Mean scores for areas of health-related quality of life (HRQOL) among colorectal cancer survivors with common bowel-related complaints.*

© 2025 Klaassen-Dekker N et al. *JAMA Network Open*.

eTable 1. *Prevalence of bowel-related complaints in colorectal cancer survivors with and without a stoma.*

Bowel-related complaints	*At 6 months after diagnosis (n=1751)*	
	**No stoma** (n=1371)	**Stoma** (n=380)
Any complaints	718 (52.4)	99 (26.1)
Diarrhea	199 (14.5)	25 (6.6)
Constipation	145 (10.6)	6 (1.6)
Flatulence/bloating	479 (34.9)	41 (10.8)
Frequent stools	341 (24.9)	25 (6.6)
Mucus in stools	76 (5.5)	18 (4.7)
False urgency	139 (10.1)	35 (9.2)
**Bowel-related complaints**	*At 2 years after diagnosis (n=1507)*	
	**No stoma** (n=1277)	**Stoma** (n=230)
Any complaints	561 (43.9)	51 (22.2)
Diarrhea	128 (10.0)	14 (6.1)
Constipation	112 (8.8)	13 (5.7)
Flatulence/bloating	398 (31.2)	33 (14.3)
Frequent stools	288 (22.6)	14 (6.1)
Mucus in stools	49 (3.8)	6 (2.6)
False urgency	70 (5.5)	5 (2.2)
**Bowel-related complaints**	*At 5 years after diagnosis (n=810)*	
	**No stoma** (n=672)	**Stoma** (n=138)
Any complaints	264 (39.3)	24 (17.4)
Diarrhea	61 (9.1)	9 (6.5)
Constipation	45 (6.7)	7 (5.1)
Flatulence/bloating	194 (28.9)	12 (8.7)
Frequent stools	134 (19.9)	5 (3.6)
Mucus in stools	29 (4.3)	4 (2.9)
False urgency	42 (6.3)	3 (2.2)

No. (%) of survivors reporting bowel-related complaints at each time point are reported in the table.

© 2025 Klaassen-Dekker N et al. *JAMA Network Open*.

eTable 2A. *Prevalence of bowel-related complaints in colorectal cancer survivors presented by type of surgery and (neo)adjuvant treatments.*

6 months after diagnosis								
	Hemicolectomy (n=615)		*Sigmoid resection (n=404)* [A]		*Low anterior resection (n=515)*			
**Bowel-related complaints**	**Surgery only** (n=396)	**Surgery + chemotherapy** (n=219)	**Surgery only** (n=245)	**Surgery + chemotherapy** (n=155)	**Surgery only** (n=197)	**Surgery + chemotherapy** (n=58)	**Surgery + radiotherapy** (n=143)	**Surgery + chemo- and radiotherapy** (n=117)
Any complaints	142 (35.9)	119 (54.3)	114 (46.5)	98 (63.2)	104 (52.8)	36 (62.1)	80 (55.9)	54 (46.2)
Diarrhea	38 (9.6)	58 (26.5)	15 (6.1)	32 (20.6)	104 (52.8)	36 (62.1)	80 (55.9)	54 (46.2)
Constipation	22 (5.6)	23 (10.5)	32 (13.1)	28 (18.1)	1 (5.6)	11 (19.0)	11 (7.7)	2 (1.7)
Flatulence/bloating	100 (25.3)	82 (37.4)	80 (32.7)	70 (45.2)	62 (31.5)	23 (39.7)	37 (25.9)	22 (18.8)
Frequent stools	39 (9.8)	35 (16.0)	47 (19.2)	44 (28.4)	68 (34.5)	18 (31.0)	59 (41.3)	31 (26.5)
Mucus in stools	9 (2.3)	6 (2.7)	6 (2.4)	6 (3.9)	13 (6.6)	4 (6.9)	25 (17.5)	14 (12.0)
False urgency	14 (3.5)	6 (2.7)	22 (9.0)	11 (7.1)	38 (19.3)	9 (15.5)	28 (19.6)	26 (22.2)
2 years after diagnosis								
**Bowel-related complaints**	**Surgery only** (n=350)	**Surgery + chemotherapy** (n=179)	**Surgery only** (n=216)	**Surgery + chemotherapy** (n=133)	**Surgery only** (n=176)	**Surgery + chemotherapy** (n=44)	**Surgery + radiotherapy** (n=126)	**Surgery + chemo- and radiotherapy** (n=99)
Any complaints	131 (37.4)	55 (30.7)	78 (36.1)	51 (38.3)	88 (50.0)	21 (47.7)	77 (61.1)	59 (59.6)
Diarrhea	46 (13.1)	18 (10.1)	7 (3.2)	5 (3.8)	18 (10.2)	3 (6.8)	15 (11.9)	19 (19.2)
Constipation	22 (6.3)	10 (5.6)	19 (8.8)	13 (9.8)	18 (10.2)	4 (9.1)	10 (7.9)	13 (13.1)
Flatulence/bloating	90 (25.7)	37 (20.7)	58 (26.9)	36 (27.1)	59 (33.5)	17 (38.6)	55 (43.7)	42 (42.4)
Frequent stools	44 (12.6)	16 (8.9)	28 (13.0)	20 (15.0)	61 (34.7)	12 (27.3)	59 (46.8)	46 (46.5)
Mucus in stools	8 (2.3)	6 (3.4)	4 (1.9)	1 (0.8)	5 (2.8)	1 (2.3)	16 (12.7)	7 (7.1)
False urgency	9 (2.6)	2 (1.1)	10 (4.6)	5 (3.8)	17 (9.7)	0 (0)	16 (12.7)	8 (8.1)
5 years after diagnosis								
**Bowel-related complaints**	**Surgery only** (n=184)	**Surgery + chemotherapy** (n=96)	**Surgery only** (n=124)	**Surgery + chemotherapy** (n=71)	**Surgery only** (n=91)	**Surgery + chemotherapy** (n=21)	**Surgery + radiotherapy** (n=72)	**Surgery + chemo- and radiotherapy** (n=63)
Any complaints	52 (28.3)	30 (31.3)	32 (25.8)	28 (39.4)	40 (44.0)	9 (42.9)	40 (55.6)	40 (63.5)
Diarrhea	18 (9.8)	11 (11.5)	5 (4.0)	2 (2.8)	10 (11.0)	2 (9.5)	9 (12.5)	9 (14.3)
Constipation	11 (6.0)	4 (4.2)	7 (5.6)	8 (11.3)	6 (6.6)	2 (9.5)	4 (5.6)	6 (9.5)
Flatulence/bloating	39 (21.2)	21 (21.9)	26 (21.0)	21 (29.6)	32 (35.2)	6 (28.6)	24 (33.3)	28 (44.4)
Frequent stools	14 (7.6)	4 (4.2)	16 (12.9)	13 (18.3)	24 (26.4)	6 (28.6)	29 (40.3)	30 (47.6)
Mucus in stools	3 (1.6)	2 (2.1)	5 (4.0)	3 (4.2)	1 (1.1)	2 (9.5)	7 (9.7)	7 (11.1)
False urgency	3 (1.6)	3 (3.1)	9 (7.3)	4 (5.6)	8 (8.8)	0 (0)	7 (9.7)	9 (14.3)

© 2025 Klaassen-Dekker N et al. *JAMA Network Open.*

eTable 2B. *Prevalence of bowel-related complaints in colorectal cancer survivors presented by type of surgery and (neo)adjuvant treatments.*

6 months after diagnosis				
	Abdominoperineal resection (n=123) [B]			*Other* [C]
**Bowel-related complaints**	**Surgery only** (n=21)	**Surgery + radiotherapy** (n=37)	**Surgery + chemo- and radiotherapy** (n=65)	**All** (n=48)
Any complaints	4 (19.0)	3 (8.1)	17 (26.2)	23 (47.9)
Diarrhea	0 (0)	1 (2.7)	4 (6.2)	7 (14.6)
Constipation	0 (0)	0 (0)	2 (3.1)	5 (10.4)
Flatulence/bloating	3 (14.3)	3 (8.1)	10 (15.4)	15 (31.3)
Frequent stools	1 (4.8)	0 (0)	4 (6.2)	11 (22.9)
Mucus in stools	0 (0)	0 (0)	2 (3.1)	3 (6.3)
False urgency	1 (4.8)	0 (0)	5 (7.7)	5 (10.4)
2 years after diagnosis				
**Bowel-related complaints**	**Surgery only** (n=20)	**Surgery + radiotherapy** (n=32)	**Surgery + chemo- and radiotherapy** (n=56)	**All** (n=41)
Any complaints	1 (5.0)	8 (25.0)	12 (21.4)	18 (43.9)
Diarrhea	0 (0)	1 (3.1)	2 (3.6)	3 (7.3)
Constipation	0 (0)	1 (3.1)	2 (3.6)	6 (14.6)
Flatulence/bloating	1 (5.0)	6 (18.8)	9 (16.1)	15 (36.6)
Frequent stools	0 (0)	1 (3.1)	3 (5.4)	5 (12.2)
Mucus in stools	0 (0)	2 (6.3)	0 (0)	2 (4.9)
False urgency	0 (0)	0 (0)	0 (0)	3 (7.3)
5 years after diagnosis				
**Bowel-related complaints**	**Surgery only** (n=10)	**Surgery + radiotherapy** (n=19)	**Surgery + chemo- and radiotherapy** (n=35)	**All** (n=20)
Any complaints	1 (10.0)	7 (36.8)	5 (14.3)	5 (25.0)
Diarrhea	0 (0)	1 (5.3)	2 (5.7)	2 (10.0)
Constipation	0 (0)	2 (10.5)	2 (5.7)	0 (0)
Flatulence/bloating	0 (0)	3 (15.8)	3 (8.6)	4 (20.0)
Frequent stools	0 (0)	0 (0)	0 (0)	3 (15.0)
Mucus in stools	0 (0)	7 (10)	0 (0)	0 (0)
False urgency	1 (10.0)	0 (0)	0 (0)	0 (0)

No. (%) of survivors reporting bowel-related complaints at each time point are reported in the table. [A] Colorectal cancer survivors who received radiotherapy (n=2) or combination of chemo- and radiotherapy (n=2) combined with a sigmoid resection were excluded due to low sample size. [B] Colorectal cancer survivors who received chemotherapy (n=3) combined with an abdominoperineal resection were excluded due to low sample size. [C] Other surgical procedures include: transversectomy (for study population at 6 months, n=19; 2 years, n=17; 5 years, n=11), subtotal colectomy (6 months, n=12; 2 years, n=10; 5 years, n=4), transanal endoscopic microsurgery (6 months, n=15; 2 years, n=12; 5 years, n=4), and other (6 months, n=2; 2 years, n=2; 5 years, n=1).

© 2025 Klaassen-Dekker N et al. *JAMA Network Open*.

eTable 3A. *Prevalence of bowel-related complaints in colorectal cancer survivors presented by type of surgery and (neo)adjuvant treatments – complete-case analysis across 795 survivors.*

6 months after diagnosis								
	Hemicolectomy (n=276)		*Sigmoid resection (n=194)* [A]		*Low anterior resection (n=243)*			
**Bowel-related complaints**	**Surgery only** (n=182)	**Surgery + chemotherapy** (n=94)	**Surgery only** (n=122)	**Surgery + chemotherapy** (n=69)	**Surgery only** (n=89)	**Surgery + chemotherapy** (n=20)	**Surgery + radiotherapy** (n=72)	**Surgery + chemo- and radiotherapy** (n=62)
Any complaints	62 (34.1)	55 (58.5)	54 (44.3)	44 (63.8)	45 (50.6)	13 (65.0)	36 (50.0)	29 (46.8)
Diarrhea	14 (7.7)	27 (28.7)	7 (5.7)	12 (17.4)	12 (13.5)	3 (15.0)	8 (11.1)	11 (17.7)
Constipation	7 (3.8)	14 (14.9)	12 (9.8)	14 (20.3)	2 (2.2)	3 (15.0)	7 (9.7)	1 (1.6)
Flatulence/bloating	44 (24.2)	42 (44.7)	39 (32.0)	33 (47.8)	28 (31.5)	8 (40.0)	13 (18.1)	17 (27.4)
Frequent stools	17 (9.3)	17 (18.1)	24 (19.7)	23 (33.3)	29 (32.6)	7 (35.0)	30 (41.7)	19 (30.6)
Mucus in stools	4 (2.2)	1 (1.1)	3 (2.5)	1 (1.4)	6 (6.7)	1 (5.0)	13 (18.1)	12 (19.4)
False urgency	6 (3.3)	1 (1.1)	13 (10.7)	4 (5.8)	18 (20.2)	5 (25.0)	13 (18.1)	13 (21.0)
2 years after diagnosis								
**Bowel-related complaints**	**Surgery only** (n=182)	**Surgery + chemotherapy** (n=94)	**Surgery only** (n=122)	**Surgery + chemotherapy** (n=69)	**Surgery only** (n=89)	**Surgery + chemotherapy** (n=20)	**Surgery + radiotherapy** (n=72)	**Surgery + chemo- and radiotherapy** (n=62)
Any complaints	67 (36.8)	29 (30.9)	46 (37.7)	26 (37.7)	40 (44.9)	10 (50.0)	45 (62.5)	37 (59.7)
Diarrhea	25 (13.7)	10 (10.6)	6 (4.9)	2 (2.9)	10 (11.2)	1 (5.0)	7 (9.7)	10 (16.1)
Constipation	11 (6.0)	6 (6.4)	11 (9.0)	5 (7.2)	5 (5.6)	2 (10.0)	5 (6.9)	6 (9.7)
Flatulence/bloating	46 (25.3)	21 (22.3)	31 (25.4)	18 (26.1)	31 (34.8)	8 (40.0)	30 (41.7)	27 (43.5)
Frequent stools	24 (13.2)	7 (7.4)	18 (14.8)	13 (18.8)	30 (33.7)	6 (30.0)	39 (54.2)	31 (50.0)
Mucus in stools	5 (2.7)	2 (2.1)	1 (0.8)	1 (1.4)	3 (3.4)	1 (5.0)	11 (15.3)	5 (8.1)
False urgency	6 (3.3)	1 (1.1)	6 (4.9)	5 (7.2)	8 (9.0)	0 (0)	13 (18.1)	4 (6.5)
5 years after diagnosis								
**Bowel-related complaints**	**Surgery only** (n=182)	**Surgery + chemotherapy** (n=94)	**Surgery only** (n=122)	**Surgery + chemotherapy** (n=69)	**Surgery only** (n=89)	**Surgery + chemotherapy** (n=20)	**Surgery + radiotherapy** (n=72)	**Surgery + chemo- and radiotherapy** (n=62)
Any complaints	52 (28.6)	29 (30.9)	32 (26.2)	27 (39.1)	39 (43.8)	9 (45.0)	40 (55.6)	40 (64.5)
Diarrhea	18 (9.9)	11 (11.7)	5 (4.1)	2 (2.9)	10 (11.2)	2 (10.0)	9 (12.5)	9 (14.5)
Constipation	11 (6.0)	4 (4.3)	7 (5.7)	7 (10.1)	6 (6.7)	2 (10.0)	4 (5.6)	6 (9.7)
Flatulence/bloating	39 (21.4)	20 (21.3)	26 (21.3)	20 (29.0)	31 (34.8)	6 (30.0)	24 (33.3)	28 (45.2)
Frequent stools	14 (7.7)	4 (4.3)	16 (13.1)	13 (18.8)	23 (25.8)	6 (30.0)	29 (40.3)	30 (48.4)
Mucus in stools	3 (1.6)	2 (2.1)	5 (4.1)	3 (4.3)	1 (1.1)	2 (10.0)	7 (9.7)	7 (11.3)
False urgency	3 (1.6)	3 (3.2)	9 (7.4)	3 (4.3)	8 (9.0)	0 (0)	7 (9.7)	9 (14.5)

© 2025 Klaassen-Dekker N et al. *JAMA Network Open.*

eTable 3B. *Prevalence of bowel-related complaints in colorectal cancer survivors presented by type of surgery and (neo)adjuvant treatments – complete-case analysis across 795 survivors.*

6 months after diagnosis				
	Abdominoperineal resection (n=62)			*Other [B]*
**Bowel-related complaints**	**Surgery only** (n=9)	**Surgery + radiotherapy** (n=18)	**Surgery + chemo- and radiotherapy** (n=35)	**All** (n=20)
Any complaints	2 (22.2)	1 (5.6)	9 (25.7)	9 (45.0)
Diarrhea	0 (0)	0 (0)	2 (5.7)	3 (15.0)
Constipation	0 (0)	0 (0)	1 (2.9)	0 (0)
Flatulence/bloating	2 (22.2)	1 (5.6)	5 (14.3)	5 (25.0)
Frequent stools	1 (11.1)	0 (0)	2 (5.7)	4 (20.0)
Mucus in stools	0 (0)	0 (0)	1 (2.9)	2 (10.0)
False urgency	0 (0)	0 (0)	2 (5.7)	0 (0)
2 years after diagnosis				
**Bowel-related complaints**	**Surgery only** (n=9)	**Surgery + radiotherapy** (n=18)	**Surgery + chemo- and radiotherapy** (n=35)	**All** (n=20)
Any complaints	0 (0)	5 (27.8)	6 (17.1)	6 (30.0)
Diarrhea	0 (0)	0 (0)	0 (0)	0 (0)
Constipation	0 (0)	1 (5.6)	1 (2.9)	1 (5.0)
Flatulence/bloating	0 (0)	4 (22.2)	5 (14.3)	5 (25.0)
Frequent stools	0 (0)	0 (0)	2 (5.7)	1 (5.0)
Mucus in stools	0 (0)	1 (5.6)	0 (0)	1 (5.0)
False urgency	0 (0)	0 (0)	0 (0)	1 (5.0)
5 years after diagnosis				
**Bowel-related complaints**	**Surgery only** (n=9)	**Surgery + radiotherapy** (n=18)	**Surgery + chemo- and radiotherapy** (n=35)	**All** (n=20)
Any complaints	1 (11.1)	7 (38.9)	5 (14.3)	5 (25.0)
Diarrhea	0 (0)	1 (5.6)	2 (5.7)	2 (10.0)
Constipation	0 (0)	2 (11.1)	2 (5.7)	0 (0)
Flatulence/bloating	0 (0)	3 (16.7)	3 (8.6)	4 (20.0)
Frequent stools	0 (0)	0 (0)	0 (0)	3 (15.0)
Mucus in stools	0 (0)	2 (11.1)	0 (0)	0 (0)
False urgency	1 (11.1)	0 (0)	0 (0)	0 (0)

No. (%) of survivors reporting bowel-related complaints at each time point are reported in the table. [A] Colorectal cancer survivors who received radiotherapy (n=1) or combination of chemo- and radiotherapy (n=2) combined with a sigmoid resection were excluded due to low sample size. [B] Other surgical procedures include: transversectomy (n=11), subtotal colectomy (n=4), transanal endoscopic microsurgery (n=4), and other (n=1).

© 2025 Klaassen-Dekker N et al. *JAMA Network Open.*

eTable 4. *Associations between dietary fiber intake and prevalence of moderate-to-severe constipation, based on data from the EORTC-QLQ-C30 questionnaire, in colorectal cancer survivors.*

**Dietary fiber intake** [A] per 10g/day at:	**Dietary fiber intake,** median (Q1-Q3), g/day	**Moderate-to-severe constipation** [B]		**Moderate-to-severe constipation** [B] adjusted for occurrence of constipation at diagnosis [C]	
		Events/total population	OR (95CI)	Events/total population	OR (95CI)
diagnosis [D]	19.7 (15.8-24.2)	159/1818	0.81 (0.55-1.19)	Not applicable	Not applicable
6 months after diagnosis [E]	19.4 (15.6-23.6)	61/1645	1.17 (0.62-2.20)	60/1627	1.20 (0.63-2.25)
2 years after diagnosis [E]	19.0 (15.0-23.7)	43/1378	0.79 (0.36-1.66)	41/1363	0.84 (0.38-1.81)
5 years after diagnosis [F]	18.5 (15.0-22.4)	22/775	0.84 (0.30-2.25)	22/767	0.82 (0.29-2.21)

[A] All models were adjusted for sex, age at diagnosis (continuous in years), tumor location (colon, rectum), and energy intake (continuous in kcal/day) at all timepoints. [B] Moderate-to-severe constipation, defined as experience of 'quite a bit' or 'very much' constipation. [C] All models are further adjusted for occurrence of moderate-to-severe constipation at diagnosis (yes/no) based on data from the EORTC-QLQ-C30 questionnaire. [D] The model at diagnosis was further adjusted for smoking status (current, former, never). [E] The model at 6 months after diagnosis was further adjusted for BMI (continuous in kg/m^2), type of treatment (surgery only, surgery + chemotherapy, surgery + radiotherapy, or surgery + chemo- and radiotherapy), and smoking status (current, former, never). [F] The model at 2 years after diagnosis was further adjusted for cancer stage (I, II, III, IV), level of moderate-to-vigorous physical activity (continuous in h/week) and smoking status (current, former, never). [F] The model at 5 years after diagnosis was further adjusted for smoking status (current, former, never). [B] All models are further adjusted for occurrence of moderate-to-severe constipation at diagnosis (yes/no) based on data from the EORTC QLQ-C30 questionnaire.

© 2025 Klaassen-Dekker N et al. *JAMA Network Open*.

eTable 5. *Associations between dietary fiber intake and prevalence of moderate-to-severe diarrhea, based on data from the EORTC-QLQ-C30 questionnaire, in colorectal cancer survivors – complete-case analysis across 690 survivors.*

**Dietary fiber intake** [A] per 10g/day at:	**Dietary fiber intake,** median (Q1-Q3), g/day	**Moderate-to-severe diarrhea** [B]		**Moderate-to-severe diarrhea** [B] adjusted for occurrence of diarrhea at diagnosis [C]	
		Events/total population	OR (95CI)	Events/total population	OR (95CI)
diagnosis	20.3 (16.3-24.6)	81/690	0.34 (0.31-0.89)	Not applicable	Not applicable
6 months after diagnosis	19.6 (16.0-23.7)	46/690	0.46 (0.22-0.92)	46/690	0.46 (0.22-0.94)
2 years after diagnosis [D]	19.6 (15.3-23.9)	23/690	0.44 (0.15-1.16)	23/690	0.44 (0.15-1.18)
5 years after diagnosis [E]	18.5 (15.2-22.4)	25/690	0.31 (0.10-0.91)	25/690	0.31 (0.10-0.91)

[A] All models were adjusted for sex, age at diagnosis (continuous in years), tumor location (colon, rectum), and energy intake (continuous in kcal/day) at all timepoints. [B] Moderate-to-severe diarrhea, defined as experience of 'quite a bit' or 'very much' diarrhea. [C] All models are further adjusted for occurrence of moderate-to-severe diarrhea at diagnosis (yes/no) based on data from the EORTC-QLQ-C30 questionnaire. [D] The model at 2 years after diagnosis was further adjusted for level of moderate-to-vigorous physical activity (continuous in h/week). [E] The model at 5 years after diagnosis was further adjusted for cancer stage (I, II, III, IV), current stoma (yes, no), BMI (continuous in kg/m^2), and smoking status (current, former, never).

© 2025 Klaassen-Dekker N et al. *JAMA Network Open*.

eTable 6. *Associations between dietary fiber intake and prevalence of moderate-to-severe constipation, based on data from the EORTC-QLQ-C30 questionnaire, in colorectal cancer survivors – complete-case analysis across 708 survivors.*

**Dietary fiber intake** [A] per 10g/day at:	**Dietary fiber intake,** median (Q1-Q3), g/day	**Moderate-to-severe constipation** [B]		**Moderate-to-severe constipation** [B] adjusted for occurrence of constipation at diagnosis [C]	
		Events/total population	OR (95CI)	Events/total population	OR (95CI)
diagnosis [D]	20.3 (16.5-24.6)	56/708	0.86 (0.47-1.54)	Not applicable	Not applicable
6 months after diagnosis [E]	19.7 (15.9-23.7)	27/708	0.99 (0.37-2.62)	27/708	0.99 (0.37-2.62)
2 years after diagnosis [E]	19.6 (15.3-23.9)	18/708	0.86 (0.25-2.78)	18/708	0.78 (0.22-2.51)
5 years after diagnosis [F]	18.5 (15.2-22.4)	22/708	0.80 (0.28-2.21)	22/708	0.77 (0.26-2.15)

[A] All models were adjusted for sex, age at diagnosis (continuous in years), tumor location (colon, rectum), and energy intake (continuous in kcal/day) at all timepoints. [B] Moderate-to-severe constipation, defined as experience of 'quite a bit' or 'very much' constipation. [C] All models are further adjusted for occurrence of moderate-to-severe constipation at diagnosis (yes/no) based on data from the EORTC-QLQ-C30 questionnaire. [D] The model at diagnosis was further adjusted for smoking status (current, former, never). [E] The model at 6 months after diagnosis was further adjusted for BMI (continuous in kg/m^2), type of treatment (surgery only, surgery + chemotherapy, surgery + radiotherapy, or surgery + chemo- and radiotherapy), and smoking status (current, former, never). [F] The model at 2 years after diagnosis was further adjusted for cancer stage (I, II, III, IV), level of moderate-to-vigorous physical activity (continuous in h/week) and smoking status (current, former, never). [F] The model at 5 years after diagnosis was further adjusted for smoking status (current, former, never). [B] All models are further adjusted for occurrence of moderate-to-severe constipation at diagnosis (yes/no) based on data from the EORTC QLQ-C30 questionnaire.

© 2025 Klaassen-Dekker N et al. *JAMA Network Open*.
