# Supplementary material for: Bowel-Related Symptoms and Dietary Fiber Intake in Colorectal Cancer Survivors
Source: JAMA Netw Open. 2025 Nov 10;8(11):e2542147. doi: 10.1001/jamanetworkopen.2025.42147 (PMC12603865; doi:10.1001/jamanetworkopen.2025.42147)
Supplement: Supplement 2. — Data Sharing Statement [file jamanetwopen-e2542147-s002.pdf]

# Data Sharing Statement

Klaassen-Dekker. Bowel-Related Symptoms and Dietary Fiber Intake in Colorectal Cancer Survivors. *JAMA Netw Open*. Published November 10, 2025.
doi:10.1001/jamanetworkopen.2025.42147

**Data**
**Data available:** No

**Additional Information**
**Explanation for why data not available:** Since the data consist of identifying patient information, some access restrictions apply and therefore data cannot be made publicly available. Data will be shared with permission from the steering committee of the COLON study. Requests for data can be sent to Dr. Dieuwertje Kok, Division of Human Nutrition and Health, Wageningen University & Research, The Netherlands (e-mail: dieuwertje.kok@wur.nl).
